# Exploring the Character Transposition Effect and Locus in Chinese Word Recognition: Evidence from Left–Right Visual Field Processing in Primary School Children

**DOI:** 10.3390/bs16020251

**Published:** 2026-02-09

**Authors:** Yi Song, Yuhan Jiang, Yuru Cheng, Lei Zhang, Jingxin Wang

**Affiliations:** 1 Faculty of Psychology, Tianjin Normal University, Tianjin 300382, China; songyi@stu.tjnu.edu.cn (Y.S.); noloft@126.com (Y.J.); cyr@stu.tjnu.edu.cn (Y.C.); fanghuohai@stu.tjnu.edu.cn (L.Z.); 2 The No. 2 Experimental Primary School of Qingdao West Coast New Area, Qingdao 266400, China

**Keywords:** letter transposition effect, character transposition effect, Chinese word recognition, visual field, hemispheric processing, position encoding, reading development

## Abstract

Prior research has offered substantial evidence for letter transposition effect in word reading, yet studies in logographic languages such as Chinese are scarce and have largely focused on adults. This study aimed to determine whether second-grade children show character transposition effect impact in recognizing two-character Chinese words and to examine potential differences between the left and right visual fields corresponding to the two cerebral hemispheres. A lexical decision task was used across two experiments. Experiment 1 tested 56 second graders and manipulated three stimulus types—normal words, Transposed pseudo-words, and Substituted pseudo-words—to verify the presence of the effect. Experiment 2 recruited an independent sample of 97 second graders and applied a lateralized presentation paradigm, presenting stimuli to either the right or left visual field (RVF/LVF), which project to the left and right hemispheres (LH/RH), respectively, to assess hemispheric differences. Experiment 1 revealed a significant character transposition effect among second-grade children. Experiment 2 showed no significant differences in the magnitude of the effect between the two visual fields. These findings provide new developmental evidence for Chinese word reading and important implications for theories of position encoding. Future studies should trace its developmental trajectory across a wider age range and diverse learning contexts.

## 1. Introduction

“Aoccdrnig to rscheearch at Cmabrigde Uinervtisy, it deosn’t mttaer in waht oredr the ltteers in a wrod are”—many people are surprised to find that they can understand this scrambled text. This everyday experience vividly illustrates a core feature of our brain’s reading system: while accurately recognizing the identity of letters (or characters), it maintains a remarkable tolerance and flexibility toward their positional information.

This leads to a central question in reading research: how does the human visual word recognition system achieve this balance? Extensive evidence from alphabetic languages has shown that when the positions of adjacent letters within a word are swapped (e.g., trail→trial), recognition efficiency is only modestly impaired—a phenomenon known as the letter transposition effect. This effect provides compelling support for the view that position encoding in word recognition is not strictly invariant (Grainger, 2008; Grainger & Whitney, 2004; Perea et al., 2021; Gomez et al., 2021).

Several theoretical models have attempted to account for the locus of this positional flexibility at different processing stages. One class of models, such as the Spatial Coding Model, attributes the ambiguity in positional information to early stages of visual perception (Davis, 2010). In contrast, another class of models, including the Open-Bigram Model, argues that the effect arises during lexical access from flexible encoding of bigram representations. Here, lexical access refers to the process of retrieving word meanings from the mental lexicon (Whitney, 2001). Therefore, the core theoretical debate centers on whether the transposition effect stems from inherent uncertainty at an early perceptual encoding stage or from flexible orthographic processing at a later lexical access stage. Subsequently, comparing how the left and right cerebral hemispheres process this effect—with the left hemisphere typically associated with fine-grained lexical analysis and the right hemisphere with holistic visual processing—can provide critical neural evidence to inform this “stage debate”.

Hemispheric differences in word processing offer a powerful approach to addressing this question. Research on functional lateralization—the phenomenon where specific cognitive functions are predominantly mediated by one cerebral hemisphere—indicates that the right hemisphere (RH)is highly sensitive to visual form and structural information of words, suggesting strong perceptual processing capabilities. In contrast, the left hemisphere (LH) shows greater sensitivity to lexical-level processing and can efficiently process linguistic stimuli with reduced cognitive demands relative to the RH (Bradshaw & Gates, 1978; Leiber, 1976; Barca et al., 2011; Chu et al., 2020; Van der Haegen et al., 2009).

For most right-handed readers, neural transmission of visual information follows a contralateral projection pattern (Gazzaniga, 2000; Corballis et al., 2002; Berlucchi, 2014), such that stimuli presented to the left visual field project to the RH (LVF/RH), whereas stimuli in the right visual field project to the LH (RVF/LH).

Thus, by presenting word stimuli separately to the left and right visual fields and comparing hemispheric differences in the transposition effect, researchers can investigate how each hemisphere contributes to the processing of positional flexibility. These insights may further inform interventions for individuals with reading difficulties, particularly those related to visual-perceptual or hemispheric integration deficits (Kirkby et al., 2022, 2025).

### 1.1. Cross-Linguistic Evidence for Transposition Effects

Research on transposition effect originates in studies of alphabetic languages. The phenomenon refers to the finding that when the positions of adjacent letters within a word are swapped, recognition difficulty increases but remains substantially lower than when the same number of letters are replaced with unrelated ones (Andrews, 1996). For example, readers are more likely to misjudge the transposed nonword jugde as the real word judge, whereas no such confusion occurs with a replacement nonword like jupte. In masked priming paradigms, transposed-letter primes (e.g., jugde) facilitate recognition of their targets (e.g., judge) significantly more than unrelated replacement-letter primes do (Perea & Lupker, 2003, 2004; Gómez et al., 2025) and corresponding to faster reaction times. In lexical decision tasks, responses to transposed pseudo-words (e.g., cholocate) tend to show lower accuracy and longer response times compared to substituted pseudo-words (e.g., choronate) (Marcet et al., 2019; Romero-Ortells et al., 2025). This phenomenon of flexible processing of positional information within alphabetic scripts is defined as the letter transposition effect (Kezilas et al., 2017; Hasenäcker & Schroeder, 2022; C. E. Lee et al., 2024; Kohnen & Castles, 2013).

In recent years, research on transposition effects has expanded beyond alphabetic scripts (writing systems that use letters to represent sounds, such as English, French, and Spanish) and has become an important avenue for evaluating the universality of reading theories. Investigators have begun to examine this phenomenon across diverse writing systems. Chinese, a logographic script with characters as its fundamental processing units, differs substantially from alphabetic scripts due to its unique combination of visuospatial complexity, tight form–meaning mappings, and the absence of interword spacing (X. Li et al., 2009). These features give rise to distinct pathways of orthographic processing and lexical access. Chinese characters, as the smallest units of free meaning (morphemes), are key nodes in lexical access. In contrast, radicals are sublexical components whose processing occurs at an earlier stage of visual feature analysis (Taft & Zhu, 1997). Additionally, investigating transposition effect requires comparing differences in reaction times and accuracy between transposition conditions and control conditions. Changing radical order typically does not produce valid characters after transposition (J. Yang, 2013), making it impossible to construct valid experimental conditions. From the perspective of controlling irrelevant variables in the experiment, using Chinese character transposition allows precise control over character frequency and visual complexity (stroke count balance). These critical variables are difficult to control at the radical level (B. Zhang & Peng, 1992). In summary, changes in Chinese character order can effectively manipulate semantics, while changes in radical order lack linguistic significance. Thus, experiment uses changes in Chinese character position information to explore character transposition effect in Chinese, which is more theoretically reasonable and methodologically feasible than using radicals as experimental materials. Gu et al. (2015) using a masked priming paradigm, demonstrated a character transposition effect in Chinese two-character word recognition—for instance, primes such as “啬吝” facilitated the recognition of their canonical forms (“吝啬”, meaning “parsimony”). H. Yang et al. (2019) further corroborated these findings by employing four-character Chinese compounds—such as “总来的说” (meaning “overall,” correctly written as “总的来说”)—and demonstrated that the effect remains stable across different text orientations. Similar research logic has also been applied in studies of Korean to investigate this effect.

Korean, by contrast, is an alphabetic language that relies more heavily on syllabic decoding than semantic extraction and is characterized by clear syllable boundaries, making the syllable a core unit of processing. C. H. Lee et al. (2015) investigated the impact of syllable transpositions on the recognition of Korean multisyllabic words, found that syllable-transposed nonwords elicited longer reaction times. Using an isolated word lexical decision task, Kim et al. (2024) examined the syllable transposition effect in Korean. They found that nonwords created by swapping the middle two syllables of a multisyllabic word (e.g., 동사무소/dong-sa-moo-so/, “Community Service Center,” → 동무사소/dong-moo-sa-so/) elicited lower accuracy and longer reaction times than replacement nonwords.

In summary, evidence from different writing systems collectively reveals a fundamental principle: positional encoding in word recognition exhibits remarkable flexibility. The transposition effect is observed not only in letter transpositions within alphabetic scripts but also in character transpositions in Chinese and syllable transpositions in Korean.

This cross-linguistic consistency challenges the notion that positional encoding must be strictly fixed and sequence-based. A current core debate centers on when this flexibility arises: whether it originates in the early perceptual encoding stage or emerges during the late lexical access process. This “stage debate” serves as a key starting point for the present study.

Empirical evidence for the transposition effect primarily comes from two experimental paradigms (Gomez et al., 2021). The first is the lexical decision task. In this paradigm, normal words, transposed pseudo-words, and substituted pseudo-words are presented randomly, and participants are required to judge their lexical status. Research shows that transposed pseudo-words (e.g., JUGDE) elicit longer reaction times and higher error rates compared to substituted pseudo-words (e.g., JUPTE). The central question addressed by this paradigm is: To what extent do transposed-letter/nonword characters generate lexical activation? The second is the masked priming paradigm. In this paradigm, a prime (which could be a normal word, a transposed pseudo-word, or a substituted pseudo-word) is presented very briefly, followed by a mask (e.g., “####”), and then a target normal word. Results typically show that the facilitatory effect from transposed pseudo-word priming on target identification is significantly greater than that from substituted pseudo-word priming. This paradigm seeks to answer: Once the orthographic representation of transposed letters/characters is pre-activated, how does it influence the subsequent processing of the target word?

In addition, nearly all existing research on transposition effect has focused on skilled adult readers, leaving a crucial developmental perspective largely unexplored. Reading acquisition is not instantaneous; its development is accompanied by substantial cognitive and neural changes. Children in the second grade are at a pivotal stage of transitioning from “learning to read” to “reading to learn” (Chall, 1983). During this period, their reading is shifting from effortful, decoding-based processing to a more automatic and consolidated stage (Ehri, 2005). Their patterns of word recognition and reading strategies undergo substantial restructuring, making this age group an ideal population for investigating developmental mechanisms such as the emergence of positional flexibility.

Examining transposition effect in children at this particular developmental stage can address a fundamental question: Is positional flexibility an inherent property of the reading system, or is it a highly efficient strategy that emerges through accumulated reading experience?

Identifying potential differences between children and adults in the manifestation of this effect will yield valuable insight into the developmental trajectory and underlying mechanisms of reading acquisition.

### 1.2. Locating the Transposition Effect

The transposition effect challenges early models that assumed strict positional encoding in word recognition (e.g., the Interactive Activation Model by McClelland & Rumelhart, 1981) and has inspired a range of new theories aiming to account for the observed positional uncertainty. Broadly, current models propose two major explanatory routes, corresponding to different processing stages.

One class of models may be termed the lexical-access account, represented by the Open-bigram Model and its derivatives (e.g., the SERIOL model: proposes that word recognition relies on the sequential temporal activation of letters from left to right; Whitney, 2001; Whitney & Cornelissen, 2008). This framework assumes that after letter identities are identified, the visual system extracts a series of ordered but non-contiguous letter pairs (e.g., “jugde” activates ju, ug, gd, de, jg, je, ue). These bigrams then activate corresponding lexical entries in the mental lexicon. Because transposed forms share a large proportion of bigrams with their base words, they strongly activate the lexical representations of the real words, leading to confusion. This account therefore places the origin of the transposition effect at a relatively late stage—lexical access.

Competing with this view is a second class of models, which may be termed the perceptual-coding account, represented by the Spatial Coding Model (Davis, 2010) and the Overlap Model (Gomez et al., 2008). These models argue that positional uncertainty arises from noise in the early, pre-lexical stage of visual processing. In this view, the position of each letter is encoded not as a precise coordinate but as a probabilistic distribution with overlap across adjacent positions. As a result, the positional signals of neighboring letters already show partial confusability at the perceptual level, and this early-stage ambiguity cascades upward to influence lexical decisions. Thus, this account locates the transposition effect at the perceptual encoding stage rather than at lexical access.

Most current visual word recognition models do not incorporate hemispheric asymmetries. The split-fovea model proposed by Monaghan and Shillcock (2008) argues that the LH encodes word input based on single-letter representations, whereas the RH uses a coarse-grained, bigram-based coding scheme. According to this model, transposed-letter stimuli (e.g., cholocate) may resemble their base words (e.g., chocolate) more when presented to the RVF (projecting to the LH) than to the LVF (projecting to the RH). Thus, the model predicts a larger likelihood of misidentifying transposed stimuli as real words under RVF/LH presentation (Perea & Fraga, 2006). The SERIOL model (Whitney, 2001; Whitney & Cornelissen, 2008; Whitney & Lavidor, 2004) also incorporates hemispheric asymmetry, assuming coarser word coding in the RH than in the LH. More recently, the PONG model (Snell, 2025) proposes that both hemispheres encode words through bigram-like representations and jointly contribute to lexical interpretation.

The lateralized presentation paradigm method has been a principal method for investigating the neural locus of specific language processes. Kim et al. (2024) provided an elegant neural-level investigation of the phenomenon through visual hemifield presentation. This technique leverages the anatomical properties of the visual pathway stimuli in the left visual field project mainly to the RH, and those in the right visual field project mainly to the LH (Gazzaniga, 2000). With brief stimulus durations to prevent eye movements, this method enables the relative isolation of each hemisphere’s processing preferences. The study found that the syllable transposition effect emerged in both visual fields, but the reaction-time effect size was larger under LVF/RH presentation. Given extensive evidence that the RH excels in holistic shape processing and visuoperceptual analysis, while the LH is dominant in fine-grained analytical and linguistic processing (Bradshaw & Gates, 1978; Chiarello, 1988), this pattern strongly suggests that the Korean syllable transposition effect is rooted at least partly in an early, perceptual stage of processing.

This finding contrasts interestingly with Perea and Fraga’s (2006) results in Spanish letter transpositions, where the effect emerged mainly in the RVF/LH condition— implying a later, lexical-access origin. Such a discrepancy may arise from differences in the basic processing units: letters are small, analytic units whose positional disturbance likely engages LH-dominant orthographic and lexical analysis; syllables are larger, more holistic perceptual units whose positional modifications may more readily be captured by RH-dominant configurational processing. These findings suggest that the cognitive and neural bases of the transposition effect are not universal but instead shaped profoundly by the structural properties of different writing systems.

### 1.3. The Present Study

Building on the theoretical background and research gaps discussed above (Whitney, 2001; Whitney & Cornelissen, 2008; Davis, 2010; Gomez et al., 2008; Gu et al., 2015; Kim et al., 2024), the present study aims to provide the first systematic investigation of the character transposition effect in second-grade Chinese children during two-character word recognition. To resolve the debate over whether the transposition effect originates at an early perceptual stage or a late lexical-access stage, it is essential not only to explore different writing systems but also to incorporate a developmental perspective. By examining second-grade children—whose orthographic knowledge is still emerging—this study leverages a developmental perspective to make stronger inferences about the locus of the transposition effect. Furthermore, by incorporating a lateralized presentation paradigm, it offers preliminary insights into hemispheric lateralization during character transposition, thereby contributing unique developmental evidence regarding its cognitive locus.

This research includes two closely connected experiments, each addressing distinct questions derived from our overarching aims.

Experiment 1:

Aim: To examine whether second-grade children exhibit a robust character transposition effect during two-character Chinese word recognition, thereby evaluating the generality of this phenomenon within a logographic writing system.

Hypothesis: In a centrally presented lexical decision task, children will show lower accuracy and longer reaction times for transposed pseudo-words (e.g., 瓜西) compared to both base words (e.g., 西瓜) and Substituted pseudo-words (e.g., 已心).

Experiment 2:

Aim: Does the magnitude of the transposition effect (operationalized as the performance difference between transposed and Substituted pseudo-words) differ between LVF/RH and RVF/LH presentation conditions?

Competing Hypotheses: (1) If the effect originates from an early perceptual stage, it should be stronger under LVF/RH presentation. (2) If the effect primarily arises at a later lexical-access stage, it should be more pronounced under RVF/LH presentation.

## 2. Experiment 1: Foveal Presentation of the Character Transposition Effect in Primary School Children

### 2.1. Method

#### 2.1.1. Participants

Fifty-six native Mandarin-speaking second-grade students participated in the study (M = 7.78 years, SD = 0.56; 29 females). All participants reported normal or corrected-to-normal vision and had no history of reading disabilities or neurological disorders. All participants were recruited from public primary schools in urban areas of a city in eastern China. These schools use the nationally standardized People’s Education Press Chinese language curriculum, ensuring consistent teaching progress in accordance with the national curriculum standards. The study was conducted in accordance with the principles of the Declaration of Helsinki. Written informed consent was obtained from the legal guardians of all participating children.

#### 2.1.2. Materials and Design

Given that two-character words constitute the most frequently used word type in modern Chinese—accounting for more than 70% of the lexicon (Wei et al., 2013)—the present study adopted two-character words as experimental stimuli. A total of 120 two-character words were selected from a standardized lexical database (Cai & Brysbaert, 2010) as base words. To balance the number of “word” and “nonword” responses, an additional 60 filler words were also selected from the same database; these fillers were not included in the data analysis. Based on the 120 base words, three stimulus conditions were constructed: (1) Normal words (180 trials in total; normally presented real words, e.g., 西瓜, “watermelon”—this condition included the 120 base words and the 60 filler words); (2) Transposed pseudo-words (120 trials; pseudo-words created by reversing the character order of the base words, e.g., 瓜西); (3) Substituted pseudo-words (120 trials; pseudo-words formed by substituting both characters of each base word with visually distinct characters, e.g., 已心).

The stimuli were divided into two blocks. Each block contained 120 Normal word trials (comprising 60 base words and 60 filler words, the filler words are identical in both blocks), 60 Transposed pseudo-word trials, and 60 Substituted pseudo-word trials. Each participant completed only one of the two blocks. Before the formal experiment, participants completed 12 practice trials using non-experimental items. Therefore, each participant completed a total of 252 experimental trials.

The three conditions (normal words, transposed pseudo-words, substituted pseudo-words) were strictly matched on first-character frequency, second-character frequency, number of strokes in each character, and total word stroke count, with no significant differences across measures (*p*s > 0.05). Substituted pseudo-words were generated by replacing both characters of a base word. The selection of replacement characters followed strict matching and control principles: (1) character structure was intentionally varied to avoid visual similarity; (2) the new combinations were verified not to constitute any existing Chinese two-character words; and (3) systematic phonological or semantic relationships with the original characters were avoided. All transposed pseudo-words were checked to ensure they were not valid Chinese words. Furthermore, any transposed pseudo-word that could potentially form a real word was excluded. The means and standard deviations for normal words and substituted pseudo-words are presented in Table 1.

#### 2.1.3. Apparatus and Procedure

Participants were tested individually in a quiet room. The experiment was programmed in E-Prime 3.0 and presented on a 16-inch Lenovo laptop. Each trial began with a central fixation cross (“+”) displayed for 500 ms. Participants were then required to decide as quickly and accurately as possible whether the subsequently presented item was a real word. They pressed F (green key) for “yes” and J (red key) for “no.” After the response, a blank screen appeared for 1000 ms, followed by the next trial. Stimuli from all conditions were randomized.

To ensure that second-grade children fully understood the task, the entire procedure was explained with the aid of pictures, and comprehension was confirmed before the formal experiment began. The experiment lasted approximately 20 min.

### 2.2. Results

Data analysis was conducted in R (R Development Core Team, 2016) using the lme4 package (Bates et al., 2014). Continuous variables were analyzed using linear mixed-effects models, whereas binary outcomes were analyzed with generalized mixed-effects models (Barr et al., 2013).

Data from all 56 participants were included, each with an overall accuracy above 80% (M = 90.97%). Accuracy for all trials and reaction times for correct trials—after removing responses shorter than 200 ms or longer than 1500 ms—are summarized in Table 2.

Linear mixed-effects model results for different word types are presented in Table 3. For overall accuracy, differences among the three-word types were also significant (substituted vs. normal: b = −3.85, z = −8.81, *p* < 0.001; transposed vs. substituted: b = −1.16, z = −4.04, *p* < 0.001), with the shortest reaction times for normal words and the longest for transposed nonwords.

For reaction times on correct trials, significant differences were observed among normal words, transposed pseudo-words, and substituted pseudo-words (substituted vs. normal: b = 118.29, t = 13.51, *p* < 0.001; transposed vs. substituted: b = 50.57, t = 4.60, *p* < 0.001), with the highest accuracy for normal words and the lowest for transposed pseudo-words.

The significant differences between transposed and substituted nonwords in both accuracy and reaction time indicate the presence of a character transposition effect (TC effect) in second-grade children during Chinese word recognition.

### 2.3. Discussion

Using a lexical decision task, the present study examined whether second-grade Chinese children exhibit a character transposition effect when recognizing two-character words. The results showed that transposed pseudo-words elicited significantly lower accuracy and longer reaction times compared with both normal words and substituted pseudo-words, demonstrating a robust character transposition effect in children’s Chinese word recognition. This pattern is highly consistent with the syllable-transposition effects observed in Korean (C. H. Lee et al., 2015; Kim et al., 2024) and the letter-transposition effects widely reported in alphabetic languages such as English (Grainger & Whitney, 2004).

The findings support the broad theoretical claim that positional encoding in visual word recognition is flexible (Davis, 2010; Gomez et al., 2008). Although Chinese characters are logographic and their recognition relies heavily on holistic processing, the present results show that altering character order substantially disrupts lexical decisions. This indicates that Chinese word recognition does not rely solely on holistic representations but also involves dynamic encoding of local positional information, whether in adults or children (Gu et al., 2015; Gu & Li, 2015).

However, while Experiment 1 confirms the presence of the character transposition effect, it does not clarify the cognitive processing stage at which this effect arises. The left hemisphere is typically associated with fine-grained lexical analysis—reflecting later stages of lexical access—whereas the right hemisphere is more involved in holistic visuospatial processing, representing earlier perceptual stages (e.g., Bradshaw & Gates, 1978; Chiarello, 1988; Leiber, 1976; Mishkin & Gorgays, 1952). Thus, a key question remains unresolved: Is the character transposition effect driven by early perceptual confusion or by later lexical-level computations of position information?

To address this issue and locate the neural locus of the effect, Experiment 2 employed a lateralized presentation paradigm, presenting stimuli either to the left visual field (projecting to the right hemisphere) or the right visual field (projecting to the left hemisphere). This design allows for dissociating hemispheric contributions and identifying the processing stage at which the effect emerges.

Overall, this study provides the first systematic evidence of the character transposition effect in Chinese-speaking children. These findings enrich cross-linguistic theories of visual word recognition and offer important insights into the cognitive mechanisms underlying Chinese reading development.

## 3. Experiment 2: Investigation of the Perceptual Location of the Character Transposition Effect in Primary School Children

### 3.1. Method

#### 3.1.1. Participants

The participants were 97 second-grade students (50 females) recruited from the same schools as in Experiment 1, with a mean age of 8.35 years (SD = 0.52). None of them had participated in Experiment 1. All participants reported normal or corrected-to-normal vision and were confirmed to be right-handed using the Edinburgh Handedness Inventory (Oldfield, 1971). None of the participants had a history of reading disorders or neurological issues. The experiment was conducted in accordance with the principles outlined in the Declaration of Helsinki. All students’ guardians provided written informed consent for participation.

#### 3.1.2. Stimuli and Experimental Design

The stimuli used in this experiment were the same as those in Experiment 1. The original two-character word materials (including base words, transposed pseudo-words, substituted pseudo-words, and filler words) were equally divided between the left and right visual fields. The experiment was divided into three blocks, each of which required the presentation of 20 base words, 20 transposed pseudo-words, 20 substituted pseudo-words, and 20 filler words for each visual field, forming a complete stimulus sequence. Each participant completed one block. As in Experiment 1, the words were matched for various lexical variables across the left and right visual field conditions. Specifically, first-character frequency, second-character frequency, number of strokes for the first and second characters, and total word stroke count were all controlled and found to have no significant differences (*p*s > 0.05). The specific matching results are shown in Table 4.

#### 3.1.3. Experimental Equipment and Procedure

The word stimuli were presented on a 16-inch (2560 × 1600 pixel) high-definition Lenovo display. The participants sat 35 cm away from the screen, with their heads positioned using a chin rest. A visual lateralization paradigm, similar to that used by Kim et al. (2024), was employed: stimuli were randomly and briefly presented to the participants’ left or right visual field. To ensure that stimuli were presented at a position 2 degrees of visual angle to either side of the parafovea, the inner edge of the two-character word was positioned 2 degrees from the central fixation point on the screen, and the outer edge was approximately 4 degrees from the central fixation point. Stimulus presentation was strictly controlled to ensure that the entire two-character string remained within a single visual hemifield.

Before the formal experiment, participants completed 16 practice trials to ensure they understood the task. During the task, participants were required to quickly and accurately determine whether the presented word was correct or incorrect, pressing the “F” key for “yes” (correct)and the “J” key for “no” (incorrect).

Before each trial, a fixation point (“+”) was presented in the center of the screen for 2000 ms. After the fixation point disappeared, the word stimulus was randomly presented to the left or to the right of the screen center for 180 ms (the brief stimulus duration was designed to prevent eye movements during word presentation). This was followed by a 3000 ms blank-screen response window. Participants were required to make their “yes” or “no” judgment during this window before the trial advanced; the trial would also terminate automatically if no response was made within the 3000 ms period. To ensure that the second-grade students understood the process, pictures were used to explain the task. The total experiment lasted approximately 25 min.

#### 3.1.4. Data Analysis

The data analysis employed linear mixed-effects models and generalized linear mixed-effects models in R, implemented using the lme4 and lmerTest packages. Both participants and items were included as crossed random effects in the models to control for the influence of individual differences and material variability on the outcomes (Baayen et al., 2008). In the linear mixed-effects models, the significance of fixed effects was assessed using t-statistics, with degrees of freedom calculated via the Satterthwaite approximation method. In the generalized linear mixed-effects models, the significance of fixed effects was tested using Wald z-statistics.

### 3.2. Results

Data were collected from 97 participants. Trials with reaction times shorter than 100 ms or greater than 2.5 standard deviations were removed. The accuracy and reaction time data for each condition are presented in Table 5.

The results of the linear mixed model for different word types are presented in Table 6. For accuracy, the main effect of word type was significant. The accuracy of transposed pseudo-words was significantly lower than that of normal words (b = −0.22, SE = 0.09, z = −2.47, *p* = 0.014). The difference between transposed pseudo-words and substituted pseudo-words was marginally significant (b = −0.21, SE = 0.12, z = −1.82, *p* = 0.069). However, the main effect of visual field was not significant (b = −0.04, SE = 0.10, z = −0.35, *p* = 0.726). More importantly, there were no significant interactions between visual field and any word type comparison (|z|s < 1.01, *p*s > 0.315), suggesting that the size of the transposition effect did not differ between the left and right visual fields.

Reaction times mirrored the accuracy pattern and showed even stronger effects. The main effect of word type was significant. Reaction times for transposed pseudo-words were significantly longer than for substituted pseudo-words (b = 0.07, SE = 0.01, t = 5.10, *p* < 0.001). Reaction times for transposed pseudo-words were also significantly longer than for normal words (b = 0.12, SE = 0.01, t = 15.85, *p* < 0.001). Consistent with the accuracy analysis, the main effect of visual field was not significant (b = 0.01, SE = 0.01, t = 1.02, *p* = 0.311). There were no significant interactions between visual field and word type in any of the comparisons (|t|s < 1.15, *p*s > 0.254).

To further confirm the non-significant interactions, simple effects analysis was conducted (see Table 7). This analysis confirmed that, for each condition (normal words, substituted pseudo-words, and transposed pseudo-words), the differences in accuracy and reaction time between the left and right visual fields were not significant (|t|s < 1.73, |z|s < 0.92, *p*s > 0.08).

### 3.3. Discussion

Experiment 2 employed a lateralized presentation paradigm to investigate the neural and cognitive locus of the character transposition effect during two-character word recognition in second-grade Chinese children. The results showed that although the character transposition effect was robust in both visual fields, no significant hemispheric differences were observed. This finding provides important clues about the processing mechanisms underlying flexible position coding in the early stages of reading development.

Unlike alphabetic scripts, where letter-transposition effects often show a left-hemisphere advantage (Perea & Fraga, 2006), or Korean syllable-transposition effects that sometimes show a right-hemisphere advantage (Kim et al., 2024), the absence of hemispheric differences in Chinese children suggests functional coordination between the two hemispheres when processing positional information. This pattern is more consistent with perceptual-encoding accounts (Davis, 2010; Gomez et al., 2008), which propose that positional uncertainty originates at early, prelexical stages of visual processing and is represented bilaterally.

It is noteworthy that although the overall character transposition effect showed no hemispheric asymmetry, real words elicited marginally faster responses in the left visual field (right hemisphere). This suggests a preliminary right-hemisphere advantage in processing holistic word forms, a pattern consistent with the right hemisphere’s specialization for visuospatial and global information processing (Bradshaw & Gates, 1978). However, this advantage disappeared when the stimuli were transposed or substituted nonwords, indicating that the left hemisphere plays an essential role in analytic orthographic processing. This condition-dependent pattern suggests that young children may rely on a flexible inter-hemispheric cooperation strategy, dynamically allocating cognitive resources based on the lexical properties of the stimuli.

Unlike alphabetic scripts composed of linearly arranged letters, each Chinese character is a compact, square-shaped visual configuration, more similar to a complex graphic pattern (J. Zhang, 2022). The right hemisphere—widely considered the “visuospatial processor” (Festini et al., 2025; Shulman et al., 2010; Corballis, 2003; Brown & Kosslyn, 1993)—is specialized for holistic shape processing, spatial relations, and complex visual patterns. Thus, when a familiar two-character word is presented as an integrated “visual pattern,” it naturally aligns with right-hemisphere processing advantages. The right hemisphere can rapidly capture its overall contour and configuration. In early reading development, children’s cognitive resources are limited, and their orthographic awareness is still emerging (H. Li et al., 2006). They tend to store newly learned words (e.g., “西瓜”) as indivisible, meaningful visual units. Therefore, when a familiar real word appears—especially briefly in the LVF/RH, the right hemisphere may quickly activate its stored visual-orthographic representation using its course, holistic encoding strategy (Monaghan & Shillcock, 2008; Kim et al., 2024), resulting in faster responses.

For nonwords, however—whether transposed (e.g., “瓜西”) or substituted (e.g., “已心”)—no stored holistic visual template exists. The right hemisphere’s rapid holistic recognition strategy therefore fails. Children must instead decompose the characters, consider their meanings and pronunciations, and evaluate their combinability. This analytic process relies more heavily on the left hemisphere (Perea & Fraga, 2006; Wang et al., 2021; Serrien & O’Regan, 2023). As a result, response times become similar between the two hemispheres for nonword conditions. This suggests that, even in early Chinese reading development, the left hemisphere already plays a critical role in fine-grained orthographic analysis. These findings have implications for early literacy instruction: at the initial stages, teaching should leverage the graphic nature of Chinese characters and strengthen children’s holistic perception and memory of word forms.

Overall, the findings depict a pattern of functional complementarity and condition-dependent lateralization: for familiar words, the right hemisphere may provide a fast, holistic recognition route; for unfamiliar strings requiring detailed analysis, the left hemisphere becomes dominant, with both hemispheres cooperating. This pattern explains why no hemispheric difference emerged for the overall character transposition effect—because the effect itself (i.e., comparing transposed vs. substituted pseudo-words) relies on contributions from both hemispheres. It depends on the right hemisphere’s initial position-uncertainty encoding as well as the left hemisphere’s final lexical-decision processes. It should be noted, however, that the absence of a significant visual field difference does not necessarily pinpoint the locus of the effect, as it may also arise from limitations in the sensitivity of the lateralized paradigm to detect hemispheric asymmetries under the current experimental conditions.

## 4. General Discussion

This study is the first to systematically investigate the presence of the Chinese character transposition effect in two-character word recognition among second-grade Chinese children by using lexical decision task, and to explore the potential brain hemisphere processing loci of this effect using lateralized presentation techniques. Our findings reveal two key conclusions: First, a robust transposition effect exists in developing Chinese readers, which confirms that the flexibility of positional encoding is already evident in the early stages of ideographic script reading development. Second, and more importantly, no significant differences were observed between the left and right visual fields (initially projecting to the right and left hemispheres, respectively), suggesting that, at the early stages of Chinese reading development, the neural basis for character position processing may be bilaterally distributed.

The stable character transposition effect observed in Experiment 1 extends evidence of the flexibility of positional encoding from alphabetic languages (e.g., Grainger & Whitney, 2004; Colombo et al., 2019; Duñabeitia et al., 2007; Lété & Fayol, 2013; Perea & Estévez, 2008) and syllabic scripts like Korean (Kim et al., 2024; C. H. Lee et al., 2015), to ideographic writing systems, particularly validating the existence of the transposition effect in the early developmental stages. The results of Experiment 1 strongly suggest that the visual word recognition system, even in its early developmental stages, does not rely on a strictly sequential or fixed positional character encoding scheme but rather employs a flexible encoding strategy that tolerates a certain degree of positional noise.

The lack of hemispheric asymmetry observed in Experiment 2 is particularly insightful. This pattern contrasts with the findings in alphabetic languages, where the transposition effect of letters has been shown to exhibit a left-hemisphere advantage (Perea & Fraga, 2006), and with recent research in Korean, where the transposition effect in syllabic scripts is stronger in the right hemisphere (Kim et al., 2024). Critically, this right-hemisphere advantage supports the perceptual-encoding account. Our bilateral pattern in Chinese children extends this, suggesting the early perceptual stage of positional encoding is fluid and bilateral in developing readers. The results of Experiment 2 suggest that, for Chinese children at this developmental stage, character positional information processing likely involves the participation of both hemispheres in a relatively balanced manner. This bilateral involvement aligns with the perceptual encoding theory of transposition effects (Davis, 2010; Gomez et al., 2008), which posits that positional uncertainty originates in the early visual-perceptual encoding phase, potentially supported by bilateral visual processing regions. If this effect were primarily driven by a later, strongly lateralized lexical access process in the left hemisphere (Bradshaw & Gates, 1978; Chiarello, 1988), we would expect to see a significant advantage in the right visual field/left hemisphere condition, but this was not observed.

The differences between our findings and those from Korean (Kim et al., 2024) or Spanish (Perea & Fraga, 2006) highlight the interaction between the characteristics of the writing system and developmental stages in shaping the neural mechanisms of orthographic processing. In contrast to Korean, where syllables as clear processing units are more easily captured by the right hemisphere’s dominant holistic/perceptual processing (Kim et al., 2024), Chinese characters, as complex visual wholes, may simultaneously engage both the right hemisphere’s overall visual-spatial processing (Monaghan & Shillcock, 2008) and the left hemisphere’s analytical processing of internal components. For developmental readers whose lexical representation and phonological skills are still consolidating (Ehri, 2005), their reading system may rely on a more distributed, bilateral network to process positional information. This balanced recruitment pattern may represent an adaptive strategy in the early stages of Chinese reading acquisition, ensuring reading system stability before strong hemisphere specialization is fully established. It is important to note that the discussion regarding the differences in the transposition effect across writing systems (e.g., the observed left-hemisphere advantage in alphabetic scripts, the right-hemisphere advantage in Korean, and the absence of a significant hemispheric difference in Chinese children in this study) and its potential links to developmental characteristics in reading is based on a theoretical interpretation and speculation of our experimental data. This speculative framework, including its developmental implications, awaits further testing in future studies involving different age groups and more ecologically valid reading contexts.

A potential methodological consideration is whether the absence of hemispheric differences could be due to insufficient perceptual or cognitive challenges. However, the significant central foveal transposition effect observed in Experiment 1 confirms that the task adequately engaged positional processing mechanisms. The use of a sensitive lexical task and well-controlled stimuli across the visual fields strengthened the credibility of the observed bilateral pattern. Additionally, base words presented in the left visual field/right hemisphere condition showed a faster marginal trend in processing, which is consistent with the right hemisphere’s role in holistic shape processing, suggesting that, if hemispheric preferences were present, the experimental paradigm used in this study would be capable of detecting them.

In conclusion, this study makes a significant contribution by confirming the existence of the character transposition effect and its bilateral neural basis in developing Chinese readers. It emphasizes the importance of considering the specific features of writing systems and developmental stages in visual word recognition theories. The findings advocate for a more nuanced developmental perspective on hemispheric functional specialization, suggesting that early reading relies on collaborative bilateral processing, which may gradually refine into more specialized lateralized patterns as expertise increases.

## 5. Limitations and Future Research Directions

This study challenge models that strictly localize positional flexibility processing to the left-hemisphere-dominated later lexical stages (e.g., Whitney, 2001). Models that allow for perceptual-level positional noise (Davis, 2010; Gomez et al., 2008) and developmental perspectives emphasizing dynamic hemispheric cooperation (e.g., Snell, 2025) are more readily accepted. The bilateral representation of the transposition effect in children suggests that positional encoding is not fixed but may evolve with reading experience.

The results offer valuable insights for early literacy instruction in Chinese. At the classroom level, educators can leverage the visual-wholistic nature of Chinese characters. Instructional activities that emphasize the overall configuration and character order of two-character words (e.g., flashcard matching, sequencing games) can foster both holistic perception and analytic processing skills in young learners. At a broader policy level, curriculum design and teacher training can incorporate an understanding of bilateral brain processing to develop teaching materials and assessment tools that balance holistic and analytic strategies. These findings may also inform the development of screening tools for identifying children at risk for reading difficulties.

Several limitations should be noted. The participant sample was limited to second-grade children, which cautions against generalizing the findings to older or adult readers. Furthermore, the study did not examine how individual differences in reading skill or cognitive abilities might modulate the observed effects. And although every effort was made to ensure hemifield-specific presentation through strict timing and positional controls, the inherent challenge of completely eliminating potential bilateral processing in lateralized paradigms remains a standard methodological consideration. Future research may employ even shorter presentation durations (e.g., 120 ms) or further adjust the parafoveal presentation angle to more thoroughly investigate the expression of this effect across visual fields. Additionally, future research could incorporate eye-tracking technology to better monitor children’s reading performance.

Future research should extend this work in several directions. Longitudinal or cross-sectional studies including a wider age range are needed to trace the developmental trajectory of the character transposition effect and its hemispheric lateralization. Employing eye-tracking technology during natural reading would provide more ecologically valid data. Investigating the moderating role of individual differences (e.g., orthographic awareness, reading fluency) is another promising avenue. Finally, neuroimaging methods could be used to directly visualize the neural networks involved in character position processing across development.

## 6. Conclusions

This study draws the following key conclusions based on two experiments:

Second-grade Chinese children exhibit a robust character transposition effect in two-character word recognition, indicating that the flexibility of positional encoding is evident in the early stages of ideographic script reading development.

The transposition effect does not show significant differences between the left and right visual fields (which project to the right and left hemispheres, respectively), suggesting that, during early development, both hemispheres may collaborate in processing character position information.

## Figures and Tables

**Table 1:** Matching of Character Frequency and Stroke Count.

	First Character Frequency	Second Character Frequency	First Character Stroke Count	Second Character Stroke Count	Total Word Stroke Count
Normal words	2079.57 (3784.98)	2235.51 (3785.67)	4.96 (1.52)	5.26 (1.95)	10.23 (2.41)
Substituted pseudo-words	2387.48 (3092.79)	2448.50 (4047.36)	4.94 (1.07)	4.88 (1.22)	9.82 (1.61)
*t*	−0.70	−0.43	0.19	1.84	1.58
*p*	0.48	0.67	0.85	0.07	0.12

Note: Values in parentheses represent standard deviations (SDs).

**Table 2:** Descriptive statistics of accuracy and reaction times across conditions.

	Normal Words	Substituted Pseudo-Words	Transposed Pseudo-Words
Accuracy (%)	99.71 (0.92)	94.78 (0.81)	88.38 (1.19)
Reaction Time (ms)	1128 (215)	1232 (177)	1278 (161)

**Table 3:** Results of Linear Mixed-Effects Models for Accuracy and Reaction Time Across Stimulus Types.

	Accuracy				Reaction Time					
	*b*	*SE*	*z*	*p*	*b*	*SE*	*t*	*p*	95% CI	
(Intercept)	4.48	0.4	11.28	<0.001	1254.1	12.71	98.64	<0.001	1229.14	1278.98
Substituted vs. Normal	−3.85	0.44	−8.81	<0.001	118.29	8.76	13.51	<0.001	101.13	135.45
Transposed vs. Substituted	−1.16	0.3	−4.04	<0.001	50.57	11.00	4.60	<0.001	29.01	72.12
Transposed vs. Normal	−5.01	0.44	−11.38	<0.001	168.86	9.02	18.73	<0.001	151.19	186.53

Note: Normal = Normal words; Transposed = Transposed pseudo-words; Substituted = Substituted pseudo-words.

**Table 4:** Matching Stroke Count and Character Frequency for Left and Right Visual Field Stimuli.

		First Character Frequency	Second Character Frequency	First Character Stroke Count	Second Character Stroke Count	Total Word Stroke Count
Normal word	left	1843.12 (2377.89)	1804.63 (3273.30)	4.83 (1.53)	5.05 (1.85)	9.88 (2.42)
	right	2387.56 (4895.10)	2710.25 (4325.30)	5.03 (1.46)	5.50 (2.08)	10.53 (2.44)
	*t*	−0.77	−1.29	−0.73	−1.25	−1.47
	*p*	0.44	0.20	0.47	0.21	0.15
Substituted Pseudo-Word	left	1949.92 (2283.38)	2901.31 (5324.93)	5.05 (0.98)	4.83 (1.12)	9.88 (1.47)
	right	2869.67 (3766.73)	2097.99 (2297.17)	4.93 (1.09)	4.88 (1.28)	9.81 (1.73)
	*t*	−1.62	1.07	0.62	−0.23	0.23
	*p*	0.11	0.29	0.54	0.82	0.83

Note: Numbers in parentheses are the standard deviations (SD).

**Table 5:** Descriptive Statistics for Accuracy and Reaction Time in Each Condition.

		Left Visual Field	Right Visual Field
Accuracy (%)	Normal	88.61 (0.82)	87.19 (0.86)
	Substituted	89.05 (0.79)	89.90 (0.76)
	Transposed	85.83 (0.89)	85.26 (0.91)
Reaction Time (ms)	Normal	1214 (12)	1228 (13)
	Substituted	968 (11)	1003 (11)
	Transposed	1299 (12)	1281 (12)

**Table 6:** Main Effects and Interactions.

	Accuracy				Reaction Time					
	*b*	*SE*	*z*	*p*	*b*	*SE*	*t*	*p*	95%CI	
(Intercept)	2.11	0.07	28.65	<0.000	6.98	0.03	267.66	<0.000	1050.41	1170.36
Left-Right	−0.04	0.10	−0.35	0.726	0.01	0.01	1.02	0.311	−12.05	35.12
Substituted vs. Normal	−0.19	0.12	−1.57	0.116	0.23	0.01	17.36	<0.000	193.27	278.45
Transposed vs. Substituted	−0.21	0.12	−1.82	0.069	0.07	0.01	5.10	<0.000	31.84	98.45
Transposed vs. Normal	−0.22	0.09	−2.47	0.014	0.12	0.01	15.85	<0.000	109.41	142.93
L-R vs. S-N	−0.24	0.24	−1.01	0.315	−0.02	0.03	−0.87	0.386	−73.51	41.4
L-R vs. S-T	0.13	0.24	0.54	0.590	−0.02	0.03	−0.59	0.555	−85.64	30.77
L-R vs. T-N	0.01	0.14	0.04	0.969	−0.02	0.02	−1.15	0.254	−57.31	9.64

Note: L-R = Left vs. Right visual field; S-N = Substituted Words vs. Normal Words; S-T = Substituted Words vs. Transposed Words; T-N = Transposed Words vs. Normal Words.

**Table 7:** Simple Effects Analysis.

	Accuracy				Reaction Time			
	*b*	*SE*	*z*	*p*	*b*	*SE*	*t*	*p*
Normal								
L-R	−0.09	0.18	−0.51	0.61	−0.03	0.02	−1.73	0.08
Substituted								
L-R	0.16	0.17	0.92	0.36	−0.01	0.02	−0.50	0.62
Transposed								
L-R	0.03	0.17	0.16	0.87	0.01	0.02	0.30	0.76

## Data Availability

The datasets generated and analyzed during the current study are not publicly available due to ethical restrictions on sharing sensitive information of minor participants but are available from the corresponding author upon reasonable request.
